# Movement behaviours are associated with lung function in middle-aged and older adults: a cross-sectional analysis of the Canadian longitudinal study on aging

**DOI:** 10.1186/s12889-018-5739-4

**Published:** 2018-07-03

**Authors:** Shilpa Dogra, Joshua Good, Matthew P. Buman, Paul A. Gardiner, Michael K. Stickland, Jennifer L. Copeland

**Affiliations:** 1 Faculty of Health Sciences (Kinesiology), University of Ontario Institute of Technology, 2000 Simcoe St N, Oshawa, ON Canada; 2 College of Health Solutions, Arizona State University, Phoenix, AZ USA; 3 Faculty of Medicine, The University of Queensland, QLD, Brisbane, Australia; 4 Faculty of Medicine and Dentistry, University of Alberta, G.F. Macdonald Centre for Lung Health, Covenant Health, Edmonton, AB Canada; 5 Department of Kinesiology & Physical Education, University of Lethbridge, 4401 University Drive, Lethbridge, AB T1K 3M4 Canada

## Abstract

**Background:**

Physical activity has been shown to attenuate the age-associated decline in lung function; however, there is little research evaluating different movement behaviours as potential correlates of lung function. Modifiable determinants need to be identified, as the prevalence of chronic respiratory disease is on the rise. The purpose of this study was to investigate associations of self-reported movement behaviours (i.e., sitting time, walking, different intensities of physical activity, and strengthening activities), with lung function in middle-aged and older adults without a respiratory disease, according to their smoking history.

**Methods:**

Data from participants of the Canadian Longitudinal Study on Aging were used for analysis (*n* = 16,839). Lung function was assessed using spirometry. A modified version of the Physical Activity Scale for the Elderly was used to assess sitting time and physical activity levels. Smoking status was classified as non-smoking, < 10 pack years smoking, and 10 or more pack years of smoking. The association between movement behaviours and lung function was assessed using hierarchical linear regression models with all covariates (age, sex, smoking status, body mass index, education, retirement status, and sleep duration) entered into block 1, and all movement behaviours entered into block 2.

**Results:**

All movement behaviours were associated with Forced Expiratory Volume in 1 s (FEV_1_) and Forced Vital Capacity (FVC) % predicted in crude and adjusted models, regardless of smoking status. Sitting time was negatively associated with both FEV_1%pred_ (β: -0.094, CI: -0.140, − 0.047) and FVC_%pred_ (β: -0.087, CI: -0.128, -0.045) among those who never smoked, and strength activity was positively associated with both FEV_1%pred_ (β: 0.272, CI: 0.048, 0.496) and FVC_%pred_ (β: 0.253, CI: 0.063,0.442) among those who smoked < 10 pack years, as well as with FVC_%pred_ among those who smoked 10 or more pack years (β: 0.309, CI: 0.064, 0.554).

**Conclusions:**

This is the first study to assess the association of different movement behaviours with lung function among middle-aged and older adults without a respiratory disease. These findings indicate that movement behaviours are correlates of lung function, and that they may be modifiable determinants of the age-associated decline in lung function.

**Electronic supplementary material:**

The online version of this article (10.1186/s12889-018-5739-4) contains supplementary material, which is available to authorized users.

## Background

Approximately 14% of adults are thought to have a mild to severe obstructive airway impairment [1], which poses a significant burden to the healthcare system [2]. In a healthy population, lung function declines with increasing age [3]. According to the World Health Organization, the number of adults over the age of 60 years is expected to double between 2015 and 2050 [4]. As such, it is critical that we identify modifiable determinants of lung function to prevent the development and progression of obstructive respiratory conditions such as chronic obstructive pulmonary disease.

Smoking is known to significantly accelerate the age-associated decline in lung function [3, 5]. Evidence suggests that despite a decrease in the number of smokers over the past several decades, the prevalence of obstructive airway impairment has remained stable when comparing data from 1988 to 1994 to data from 2007 to 2010 [1]. Clearly there are additional factors beyond smoking status that influence the observed age-associated decline in lung function. Although limited research is available, recent evidence suggests that both light intensity physical activity [6], and prolonged sitting time are associated with a variety of health outcomes [7], including lung function [8, 9]. Furthermore, research indicates that moderate to high levels of physical activity may attenuate the accelerated decline in lung function associated with smoking [10]. However, there is no research to date investigating the association between varying intensities of physical activity, or strength training activity with lung function measures.

Understanding the effect of movement behaviours on lung function may be especially important in an older population, as increasing age is associated with lower physical activity participation and higher volumes of prolonged sitting time [11, 12]. While individuals may be physically active, that is, meeting minimum recommendations of 150 min of moderate intensity physical activity per week and/or participating in strengthening activities twice per week, they may also be accumulating a large volume of sedentary time by sitting for prolonged periods during recreation, workplace, household, and transportation activities. Understanding the association of different movement behaviours with lung function would shed light on intervention targets that could significantly influence the age-associated decline in lung function among smokers and non-smokers. Thus, the purpose of this study was to investigate the association of movement behaviours (i.e. sitting time, walking, different intensities of physical activity, and strengthening activities) and lung function in middle-aged and older males and females without a respiratory disease, according to their smoking history.

## Methods

### Data source and participants

The Canadian Longitudinal Study on Aging (CLSA) is a nationally representative, stratified, random sample of 51,338 Canadian women and men aged 45 to 85 years (at baseline). The purpose of this survey is to collect data on the health and quality of life of Canadians to better understand the processes and dimensions of aging. The CLSA participants were selected randomly for inclusion. The study contains two samples; the CLSA Comprehensive and the CLSA Tracking. Data from participants in the first sample were collected through questionnaires, physical examinations and biological samples. These participants live within a 25-50 km radius of one of the 11 data collection sites across Canada (Vancouver/Surrey (two sites), Victoria, Calgary, Winnipeg, Hamilton, Ottawa, Montreal, Sherbrooke, Halifax, and St. John’s). This sample contains approximately 30,000 participants, recruited between 2012 and 2015, and was used for the proposed research.

Inclusion in the CLSA was limited to those who were able to read and speak either French or English. Residents in the three territories and some remote regions, persons living on federal First Nations reserves and other First Nations settlements in the provinces, and full-time members of the Canadian Armed Forces were excluded. Individuals living in long-term care institutions (i.e., those providing 24-h nursing care) were excluded at baseline; however, those living in households and transitional housing arrangements (e.g., seniors’ residences, in which only minimal care is provided) were included. Finally, those with a cognitive impairment at the time of recruitment were excluded.

For the present analysis, those with self-reported obstructive airway disease and/or lung cancer were excluded (*n* = 24,744); an analysis on this group is available elsewhere [13]. Only those who had complete data for lung function (*n* = 17,783), physical activity and sitting time (n = 17,004), and all covariates (*n* = 16,839) were included.

### Measures

#### Lung function

Spirometry was conducted using the TruFlow Easy-On Spirometer. Only those with major contraindications did not perform the test. Maximal inspiratory and expiratory maneuvers were performed to obtain Forced Expiratory Volume in 1 s (FEV_1_) and Forced Vital Capacity (FVC) values. Each participant’s data were graded to indicate repeatability. Further detail on the procedures can be found in the CLSA spirometry standard operating procedures [14].

The best FEV_1_ and best FVC measures of participants were used for analysis; participants who were unable to complete three acceptable tests were excluded. Participants with data outside of normal physiological limits were also excluded (FEV_1_ or FVC > 10 Litres). Sex and age-specific formulas developed on the Canadian population were used to determine percent of predicted FEV_1_ (FEV_1%pred_) and FVC (FVC_%pred_) [15].

#### Physical activity and sitting time

A modified version of the Physical Activity Scale for Elderly (PASE) was used to collect information on sitting time and physical activity. The PASE is a valid and reliable tool for measuring physical activity and sitting time among older adults. It has been shown to have good test-retest reliability over a 3 to 7-week interval (0.75, 95% CI = 0.69–0.80). Construct validity has also been established [16].

With regards to *sitting time* specifically, participants were asked “Over the past 7 days, how often did you participate in sitting activities such as reading, watching TV, computer activities or doing handicrafts?” and “On average, how many hours per day did you engage in these sitting activities?”. The frequency of individual sitting activities was recorded in categories of never, seldom (1 to 2 days), sometimes (3 to 4 days), or often (5 to 7 days) for frequency, and the duration of individual sitting activities was recorded in categories of < 30 min, 30 min to < 1 h, 1 h to < 2 h, 2 h to < 4 h, or 4 h or more. The midpoint of each frequency and duration category (except for the 4 h or more hours category, which was coded as 4 h), was used to estimate weekly total sitting time in hours per week.

The PASE also asks a series of questions pertaining to physical activity over the past 7 days. Specifically, participants were asked how often they took a walk outside (*walking*), engaged in *light* sports or recreational activities, engaged in *moderate* sports or recreational activities, engaged in *strenuous* sports or recreational activities, and engaged in exercises specifically to increase muscle strength and endurance (*strengthening*). The frequency and duration for each activity was recorded in the same way as for sitting time; the same midpoints were used to calculate hours per week spent in each type /intensity of activity.

#### Smoking status

Pack years were calculated using eight variables from the CLSA. Participants who responded negatively to “Have you smoked at least 100 cigarettes in your life? (about 4 - 5 packs)” were categorized as “Never Smoked”. Participants who were current smokers were asked “For how many total years have you smoked daily?” and “During the total years that you have smoked daily, about how many cigarettes per day have you usually smoked? (If your smoking pattern has changed over the years, make your best guess of the average number of cigarettes you have smoked per day.)” The number of cigarettes smoked per day was recorded in categories of 1–5, 6–10, 11–15, 16–20, 21–25, and 26+ cigarettes. The midpoint of each of category was used to determine the number of cigarettes smoked per day with the exception of 26+ cigarettes in which case an exact number was recorded. Similar questions were asked to former daily smokers.

Pack years was calculated as: [(number of cigarettes smoked per day/20 cigarettes per pack) x number of years smoked]. This was then categorized into Never Smoked, < 10 pack years, and 10 or more pack years. Participants who were never daily smokers but had smoked more than 100 lifetime cigarettes were included in the < 10 pack years category.

#### Covariates

Participants were asked to report their age and sex, and provided responses to several relevant covariates. For *sleep*, participants were asked “During the past month, on average, how many hours of actual sleep did you get at night?”. This was categorized into < 6 h, 6–8 h, and > 8 h according to previous research on the association between sleep and health [17]. For *retirement status*, participants were asked “At this time, do you consider yourself to be completely retired, partly retired or not retired”. Those who responded partly retired were merged with the not retired group due to sample size. For *education levels*, participants were asked four questions pertaining to their highest level of education. These responses were combined to categorize the sample as: Less than secondary school graduation, secondary school graduation (no post-secondary education), some post-secondary education, or post-secondary degree/diploma. Height and weight were measured by trained professionals, and used to calculate *body mass index* (kg/m^2^).

Full standard operating procedures and questionnaires for all variables analyzed can be found on the CLSA website [18].

### Statistical analysis

Means and frequencies were used to describe the sample. One way analysis of variance and post hoc pairwise comparisons were used to detect differences in continuous sample characteristics between respondents in different smoking status groups. Separate tests were run for males and females. Chi-square tests were used to detect differences in categorical sample characteristics, for males and females separately.

Crude associations between lung function (FEV_1%pred_ or FVC_%pred_) and movement behaviours (sitting time, walking, light physical activity, moderate physical activity, strenuous physical activity, and muscle strengthening activity) were assessed using linear regression models.

Hierarchical models were used to generate adjusted associations. Specifically, block 1 contained all of the covariates while block 2 included each of the movement behaviours. Models were run separately for each smoking status group, and separately for males and females (Additional files 1, 2, 3 and 4).

All statistics were performed using SPSS v.24. To ensure national representation and to compensate for under-represented groups, sampling weights were applied to regression models. Additional details on sampling, methods and weighting on the CLSA can be found in the protocol document [19, 20].

## Results

The overall sample was 62.0 ± 10.0 years of age, with 49.1% being males; 49.9% never smoked, 25.8% had less than 10 pack years, and 24.3% had 10 or more pack years smoking history. Participants averaged 17.8 ± 6.2 h of sitting time, 4.5 ± 4.6 h of walking, 0.9 ± 2.6 h of light intensity physical activity, 0.8 ± 2.7 h of moderate intensity physical activity, 1.6 ± 3.2 h of strenuous intensity physical activity, and 0.7 ± 1.7 h of strengthening activity, per week. The average FEV_1%pred_ was 98.4 ± 15.7% and FVC_%pred_ was 93.5 ± 13.6%. Additional sample characteristics by sex and smoking status can be found in Table 1. Chi-square values for all categorical variables (except for retirement status in females) were significant, indicating that education, retirement status, and sleep differed by smoking status within the male and female groups.

**Table 1:** Sample characteristics according to smoking history and sex

Characteristics		Never Smoked		< 10 Pack Years		10 or more Pack Years	
		Males (*n* = 3872)	Females (*n* = 4540)	Males (*n* = 2067)	Females (*n* = 2278)	Males (*n* = 2328)	Females (*n* = 1756)
Age (years)		60.7 ± 10.0 ^a,b^	62.0 ± 10.3	62.1 ± 9.9 ^a,c^	61.6 ± 9.9	64.4 ± 9.5 ^b,c^	62.3 ± 9.4
BMI (kg/m^2^)		27.9 ± 4.6 ^b^	27.0 ± 5.5 ^b^	27.9 ± 4.1 ^c^	27.2 ± 5.3 ^c^	28.9 ± 4.8 ^b,c^	28.2 ± 6.1 ^b,c^
Education (% of sample)	Less than secondary school graduation	2.0%	3.7%	3.1%	4.2%	7.4%	7.6%
	Secondary school graduation, no post-secondary education	5.7%	8.8%	8.4%	9.5%	11.3%	15.4%
	Some post-secondary education	5.8%	6.1%	7.1%	8.1%	9.5%	9.8%
	Post-secondary degree/diploma	86.5%	81.5%	81.4%	78.2%	71.8%	67.1%
Retirement Status	Retired	33.1%	43.5%	37.6%	43.8%	47.0%	45.4%
	Not or partly retired	66.9%	56.5%	62.4%	56.2%	53.0%	54.6%
Activity Levels (hours/week)	In sitting activities	17.4 ± 6.3 ^b^	17.5 ± 6.3 ^b^	17.4 ± 6.2 ^c^	17.8 ± 6.3 ^c^	18.6 ± 5.8 ^b,c^	18.6 ± 5.9 ^b,c^
	Walking	4.3 ± 4.6 ^a^	4.4 ± 4.5	4.7 ± 4.7 ^a^	4.6 ± 4.6	4.5 ± 5.0	4.3 ± 4.5
	Light activities	0.9 ± 2.8	0.9 ± 2.3	1.0 ± 3.0	0.8 ± 2.3	1.0 ± 2.9	0.8 ± 2.5
	Moderate sports or recreational activities	0.9 ± 3.0	0.6 ± 2.2	1.1 ± 3.3	0.7 ± 2.3	0.9 ± 3.2	0.7 ± 2.3
	Strenuous sports or recreational activities	1.9 ± 3.4 ^b^	1.4 ± 3.0 ^b^	2.0 ± 3.7 ^c^	1.5 ± 3.1 ^c^	1.5 ± 3.4 ^b,c^	0.9 ± 2.3 ^b,c^
	Increase muscle strength and endurance	0.8 ± 1.8 ^b^	0.6 ± 1.5	0.9 ± 1.9 ^c^	0.7 ± 1.7 ^c^	0.7 ± 1.8 ^b,c^	0.6 ± 1.5 ^c^
Sleep	Less than 6 h	9.5%	12.0%	10.1%	12.8%	12.3%	14.2%
	6 to 8 h	87.1%	82.8%	86.2%	80.3%	82.6%	78.8%
	More than 8 h	3.4%	5.1%	3.8%	6.9%	5.1%	7.0%
FEV_1_ (L)		3.4 ± 0.7 ^b^	2.4 ± 0.5 ^a,b^	3.3 ± 0.7 ^c^	2.4 ± 0.5 ^a,c^	3.0 ± 0.7 ^b,c^	2.2 ± 0.5 ^b,c^
FVC (L)		4.3 ± 0.8 ^b^	3.0 ± 0.6 ^a,b^	4.3 ± 0.8 ^c^	3.1 ± 0.6 ^a,c^	4.0 ± 0.8 ^b,c^	2.9 ± 0.6 ^b,c^
FEV_1_% predicted		98.6 ± 14.6 ^b^	101.0 ± 15.7 ^b^	99.3 ± 14.8 ^c^	101.3 ± 15.1 ^c^	92.4 ± ^b,c^ 16.2	94.9 ± 16.1 ^b,c^
FVC % predicted		92.8 ± 13.1 ^b^	95.8 ± 14.1 ^a,b^	93.5 ± 12.6 ^c^	96.8 ± 13.1 ^a,c^	88.3 ± 13.5 ^b,c^	92.5 ± 13.5 ^b,c^

^a^*p* < 0.05 for Never Smoked vs. < 10 pack years

^b^*p* < 0.05 for Never Smoked vs. 10 or more pack years

^c^*p* < 0.05 for < 10 pack years vs. 10 or more pack years

**Fig. 1:**
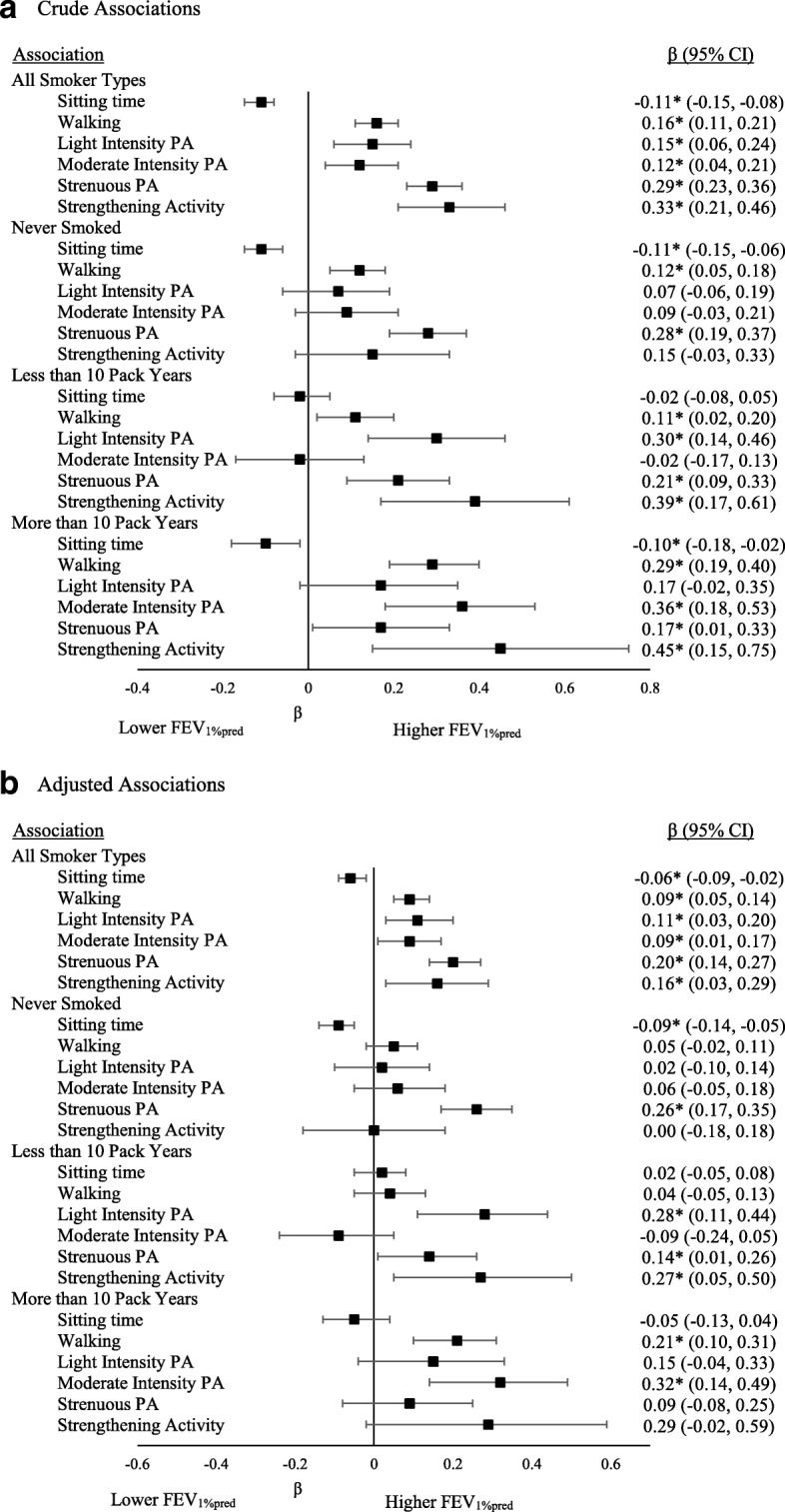
Associations between movement behaviours and FEV_1%pred_ by smoking history. **a**. Crude Associations. **b**. Adjusted Associations

**Fig. 2:**
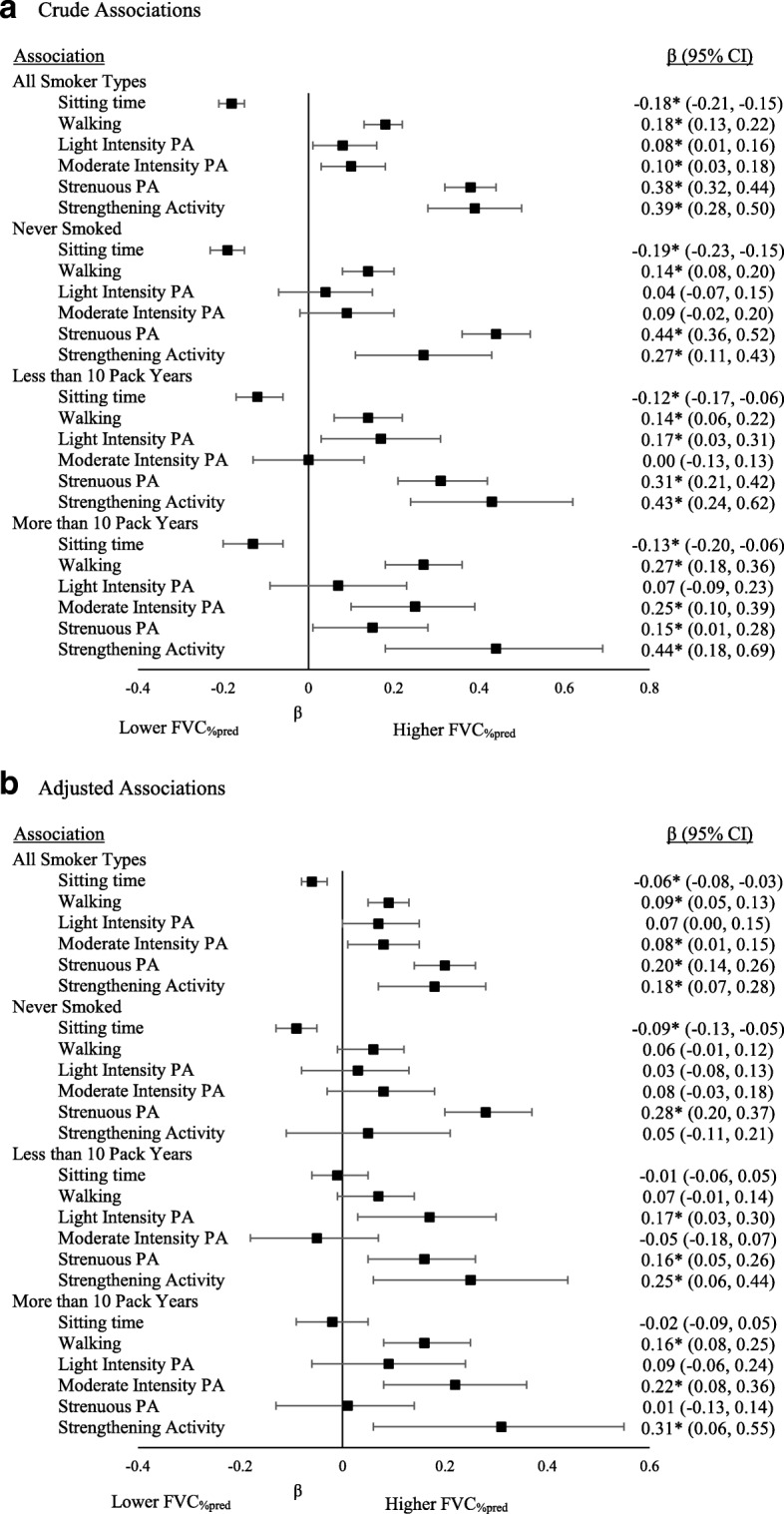
Associations between movement behaviours and FVC_%pred_ by smoking history. **a**. Crude Associations. **b**. Adjusted Associations

The associations between FEV_1%pred_ and movement behaviours are presented in Fig. 1. In analyses containing all smoking status groups, crude and adjusted associations between FEV_1%pred_ and all movement behaviours were significant. This model explained 4.7% of the variance in FEV_1%pred_. The associations between FVC_%pred_ and movement behaviours are presented in Fig. 2. In analyses containing all smoking status groups, crude and adjusted associations between FVC_%pred_ and all movement behaviours (except for light intensity physical activity in the adjusted model (β: 0.074, CI: 0.000, 0.147)) were significant. This model explained 10.5% of the variance in FVC_%pred._

Among those who never smoked, the association of FEV_1%pred_ and FVC_%pred_ with sitting time and strenuous intensity physical activity remained significant in adjusted models such that lower sitting time and higher strenuous physical activity levels were associated with better lung function. Among those who smoked less than 10 pack years, there was a significant positive association of FEV_1%pred_ and FVC_%pred_ with light intensity, strenuous intensity, and strengthening activity in adjusted models. Among those who smoked more than 10 pack years, there was a positive association of FEV_1%pred_ and FVC_%pred_ with walking and moderate intensity physical activity in adjusted models. For FVC_%pred_, there was also an association with strengthening activity among those who smoked more than 10 pack years.

For both FEV_1%pred_ and FVC_%pred_, the addition of movement behaviours to block 2 led to a statistically significant increase in R^2^. When stratified by sex, some differences were observed, however, the differences were not consistent; separate associations for males and females are shown in Additional files 1, 2, 3 and 4.

## Discussion

Using data from the CLSA, we sought to determine associations of different movement behaviours with lung function among middle-aged and older adults who did not report a respiratory disease. Our primary finding is that time spent sitting, walking, in different intensities of physical activity, and in strengthening activity are all associated with FEV_1%pred_ and FVC_%pred_ regardless of smoking history after adjusting for several covariates. In other words, less weekly sitting time and more weekly physical activity were associated with better lung function. The effect of each individual movement behaviour was small; thus, future research is needed to evaluate the cumulative effect of changing all behaviours on lung function. Importantly, the addition of movement behaviours to our models significantly increased the amount of variance explained in FEV_1%pred_ and FVC_%pred_. Together, these findings indicate that movement behaviours are correlates of lung function, and may be modifiable determinants of the age-associated decline in lung function.

Sitting time was negatively associated with FEV_1%pred_ and FVC_%pred_ in crude and adjusted models that included all smoker groups, such that lower sitting time was associated with higher FEV_1%pred_ and higher FVC_%pred_. Our findings are consistent with a previous study of adults aged 45–74 years that found a negative association between hours of TV viewing and FEV_1_ after adjusting for smoking status (never, former, current) in their analysis [8]. Similarly, a study using a younger sample found significant associations for FVC and sedentary behaviour among boys; however, no other consistent associations were observed, likely due to the young age of their sample [9]. It is possible that excessive sitting time accelerates the age-associated decline in lung function. The decline in lung function is associated with systemic inflammation [21, 22], and sitting time is known to be associated with markers of chronic low-grade inflammation [23, 24]. Thus it is possible that inflammation is a biological link between prolonged sitting time and declining lung function. Future research using longitudinal data is needed to better understand the rate of decline, and the interactions with smoking history.

Walking as well as light, moderate, and strenuous intensity physical activity were associated with FEV_1%pred_ in crude and adjusted models, regardless of smoking status. All movement behaviours but light intensity physical activity were also associated with FVC_%pred_ in crude and adjusted models, regardless of smoking status. These results were not surprising since previous literature indicates that higher cardiorespiratory fitness is associated with less of a decline in lung function [25], that athletes have better lung function than sedentary controls [26], that among middle-aged to older smokers, higher physical activity levels are associated with better lung function [27], and that stair climbing and vigorous intensity physical activity are associated with higher FEV_1_ among middle-aged and older adults [8]. Furthermore, data from the Canadian Health Measures Survey indicates that among adults aged 40–79 years, the association of waist circumference with FEV_1_ and FVC is confounded by physical activity and physical fitness [28]. As with sedentary time, inflammation may mediate the relationship between physical activity and lung function, as cardiorespiratory fitness and physical activity are independently associated with lower levels of inflammation [29]. Evidence of an association between physical activity and lung function is building; longitudinal work is needed to better understand this relationship.

To our knowledge, this is the first study to examine the association between participation in strengthening activity and lung function. The association was significant in crude and adjusted models when all smoking status groups were in the model, as well as in models containing those with a history of smoking (< 10 pack years and 10 or more pack years), but not among those who never smoked. This is consistent with meta-analytic data showing that among those with diagnosed chronic respiratory conditions, strength training improves several respiratory outcomes, including FEV_1_ [30]. Thus, it is plausible that strength training has more of an effect on lung function among those with a smoking history than it would among those who have never smoked. Research is needed to determine the influence of volume and intensity of strengthening activity on lung function.

Differences were also observed for other movement behaviours between the groups based on smoking history. Among those who never smoked, sitting time and strenuous intensity activity were associated with FEV_1%pred_ and FVC_%pred_; among those who had a history of less than 10 pack years, light and strenuous activity were associated with FEV_1%pred_ and FVC_%pred_; and among those who had 10 or more pack years smoking history, walking and moderate intensity activity were associated with FEV_1%pred_ and FVC_%pred_. It is difficult to determine if these differences are truly related to the intensity and type of activity, or if the differences are related to sample size and variability. Future research assessing the association between measured cardiorespiratory fitness and/or musculoskeletal fitness with lung function is needed, as ultimately, the movement behaviours collectively affect fitness, and fitness is a strong predictor of most health outcomes [31–33].

Strengths of this study include the large representative sample, the lung function measures, and use of a validated questionnaire for sitting time and physical activity. Nevertheless, device-measured movement behaviours would provide more valid data, as well as the opportunity to analyse differences between prolonged and interrupted sitting time with lung function. Another limitation is the self-reported smoking history. It is possible that participants were misclassified based on our calculated variable of pack years. Another limitation is that the FVC appears to be underestimated as the mean FVC_%pred_ in the overall CLSA cohort was 93%. Although the FVC data were reproducible, the reduced FVC suggests that participants may not have exhaled for the maximal possible duration. Finally, it is important to note that data from the CLSA are cross-sectional, thus, reverse-causality cannot be ruled out at this time. This is of critical importance given that lung function declines with age, and that lower lung function leads to shortness of breath, deconditioning, and a subsequent decline in physical activity levels. Thus, it is not clear whether movement behaviours change as a result of declining lung function, or whether low levels of activity accelerate the age-associated decline in lung function. In the next 5 years, the CLSA will provide its first round of longitudinal data where these associations can be further analyzed.

## Conclusion

In conclusion, among a sample of Canadian adults aged 45 years and older without respiratory disease, sitting time, walking, light, moderate, and strenuous intensity physical activity, and strengthening activity were associated with measures of lung function (FEV_1%pred_ and FVC_%pred_) regardless of smoking history. This study is the first to our knowledge to conduct a comprehensive assessment of the association between lung function and movement behaviours, and provides important information for future research. Movement behaviours may be modifiable determinants of lung function, and could be targeted to attenuate the age-associated decline in lung function.

## Additional files

Additional file 1: **Table S1.** Association between movement behaviours and %predicted FEV_1_ by smoking history among males. (DOCX 21 kb)

Additional file 2: **Table S2.** Association between movement behaviours and %predicted FEV_1_ by smoking history among females. (DOCX 21 kb)

Additional file 3: **Table S3.** Association between movement behaviours and %predicted FVC by smoking history among males. (DOCX 22 kb)

Additional file 4: **Table S4.** Association between movement behaviours and %predicted FVC by smoking history among females. (DOCX 22 kb)

## Abbreviations

CI: Confidence Interval
CLSA: Canadian Longitudinal Study on Aging
FEV_1_: Forced Expiratory Volume in One Second
FEV_1%pred_: Percent of predicted Forced Expiratory Volume in One Second
FVC: Forced Vital Capacity
FVC_%pred_: Percent of predicted Forced Vital Capacity
PASE: Physical Activity Scale for Elderly
