# Supplementary material for: Movement behaviours are associated with lung function in middle-aged and older adults: a cross-sectional analysis of the Canadian longitudinal study on aging
Source: BMC Public Health. 2018 Jul 3;18:818. doi: 10.1186/s12889-018-5739-4 (PMC6029121; doi:10.1186/s12889-018-5739-4)
Supplement: Supplementary file 1 — Table S1. Association between movement behaviours and %predicted FEV1 by smoking history among males. (DOCX 21 kb) [file 12889_2018_5739_MOESM1_ESM.docx]

Additional file

Table S1: Association between movement behaviours and %predicted FEV_1_ by smoking history among males

a) All Smoker Types

|  | **Crude Associations** | | | | **Adjusted Associations** | | | |
| --- | --- | --- | --- | --- | --- | --- | --- | --- |
| **Variable** | **R^2^** | **ẞ** | **CI** | **R^2^** | | **ẞ** | **CI** |  |
| Sitting Time (hours/week) | 0.005 | -0.162* | (-0.211, -0.113) | 0.063 | | -0.076* | (-0.125, -0.027) |  |
| Walking (hours/week) | 0.003 | 0.157* | (0.092, 0.223) |  |  | 0.100* | (0.036, 0.164) |  |
| Light Intensity PA (hours/week) | 0.001 | 0.165* | (0.053, 0.276) |  |  | 0.156* | (0.046, 0.265) |  |
| Moderate Intensity PA (hours/week) | 0.001 | 0.174* | (0.074, 0.273) |  |  | 0.130* | (0.032, 0.227) |  |
| Strenuous PA (hours/week) | 0.008 | 0.366* | (0.281, 0.451) |  |  | 0.213* | (0.128, 0.299) |  |
| Strengthening Activity (hours/week) | 0.003 | 0.438* | (0.271, 0.605) |  |  | 0.210* | (0.044, 0.376) |  |

*The R^2^ for Block 1 was 0.054. This increased significantly when adding Block 2 (p<0.001).*

**p<0.05*

b) Never smoked

|  | **Crude Associations** | | | **Adjusted Associations** | | |
| --- | --- | --- | --- | --- | --- | --- |
| **Variable** | **R^2^** | **ẞ** | **CI** | **R^2^** | **ẞ** | **CI** |
| Sitting Time (hours/week) | 0.003 | -0.127* | (-0.191, -0.063) | 0.051 | -0.072* | (-0.137, -0.008) |
| Walking (hours/week) | 0.001 | 0.098* | (0.008, 0.187) |  | 0.046 | (-0.043, 0.135) |
| Light Intensity PA (hours/week) | 0.000 | 0.081 | (-0.077, 0.237) |  | 0.088 | (-0.066, 0.242) |
| Moderate Intensity PA (hours/week) | 0.001 | 0.125 | (-0.018, 0.268) |  | 0.110 | (-0.031, 0.251) |
| Strenuous PA (hours/week) | 0.011 | 0.398* | (0.283, 0.513) |  | 0.304* | (0.188, 0.419) |
| Strengthening Activity (hours/week) | 0.001 | 0.213 | (-0.019, 0.446) |  | 0.008 | (-0.222, 0.238) |

*The R^2^ for Block 1 was 0.042. This increased significantly when adding Block 2 (p<0.001).*

**p<0.05*

c) Less than 10 pack years

|  | **Crude Associations** | | | **Adjusted Associations** | | |
| --- | --- | --- | --- | --- | --- | --- |
| **Variable** | **R^2^** | **ẞ** | **CI** | **R^2^** | **ẞ** | **CI** |
| Sitting Time (hours/week) | 0.001 | -0.084 | (-0.179, 0.012) | 0.027 | -0.036 | (-0.132, 0.061) |
| Walking (hours/week) | 0.000 | -0.032 | (-0.157, 0.094) |  | -0.083 | (-0.209, 0.044) |
| Light Intensity PA (hours/week) | 0.002 | 0.234* | (0.032, 0.436) |  | 0.232* | (0.029, 0.436) |
| Moderate Intensity PA (hours/week) | 0.000 | -0.058 | (-0.233, 0.116) |  | -0.138 | (-0.314, 0.039) |
| Strenuous PA (hours/week) | 0.005 | 0.271* | (0.112, 0.430) |  | 0.176* | (0.010, 0.342) |
| Strengthening Activity (hours/week) | 0.003 | 0.387* | (0.097, 0.676) |  | 0.302 | (-0.002, 0.606) |

*The R^2^ for Block 1 was 0.018. This increased significantly when adding Block 2 (p=0.004).*

**p<0.05*

d) More than 10 pack years

|  | **Crude Associations** | | | **Adjusted Associations** | | |
| --- | --- | --- | --- | --- | --- | --- |
| **Variable** | **R^2^** | **ẞ** | **CI** | **R^2^** | **ẞ** | **CI** |
| Sitting Time (hours/week) | 0.004 | -0.178* | (-0.294, -0.063) | 0.069 | -0.092 | (-0.207, 0.023) |
| Walking (hours/week) | 0.020 | 0.441* | (0.308, 0.573) |  | 0.331* | (0.198, 0.463) |
| Light Intensity PA (hours/week) | 0.002 | 0.253* | (0.015, 0.491) |  | 0.247* | (0.014, 0.479) |
| Moderate Intensity PA (hours/week) | 0.011 | 0.506* | (0.298, 0.714) |  | 0.436* | (0.232, 0.640) |
| Strenuous PA (hours/week) | 0.002 | 0.195* | (0.008, 0.381) |  | 0.037 | (-0.148, 0.223) |
| Strengthening Activity (hours/week) | 0.006 | 0.699* | (0.321, 1.076) |  | 0.478* | (0.102, 0.854) |

*The R^2^ for Block 1 was 0.038. This increased significantly when adding Block 2 (p<0.001).*

**p<0.05*
