# Supplementary material for: Movement behaviours are associated with lung function in middle-aged and older adults: a cross-sectional analysis of the Canadian longitudinal study on aging
Source: BMC Public Health. 2018 Jul 3;18:818. doi: 10.1186/s12889-018-5739-4 (PMC6029121; doi:10.1186/s12889-018-5739-4)
Supplement: Supplementary file 2 — Table S2. Association between movement behaviours and %predicted FEV1 by smoking history among females. (DOCX 21 kb) [file 12889_2018_5739_MOESM2_ESM.docx]

Additional file

Table S2: Association between movement behaviours and %predicted FEV_1_ by smoking history among females

a) All Smoker Types

|  | **Crude Associations** | | | | **Adjusted Associations** | | | |
| --- | --- | --- | --- | --- | --- | --- | --- | --- |
| **Variable** | **R^2^** | **ẞ** | **CI** | **R^2^** | | **ẞ** | **CI** |  |
| Sitting Time (hours/week) | 0.001 | -0.065* | (-0.114, -0.015) | 0.030 | | -0.042 | (-0.092, 0.008) |  |
| Walking (hours/week) | 0.002 | 0.158* | (0.087, 0.228) |  |  | 0.098* | (0.027, 0.169) |  |
| Light Intensity PA (hours/week) | 0.000 | 0.145* | (0.007, 0.283) |  |  | 0.066 | (-0.071, 0.203) |  |
| Moderate Intensity PA (hours/week) | 0.000 | 0.108 | (-0.036, 0.252) |  |  | 0.026 | (-0.117, 0.169) |  |
| Strenuous PA (hours/week) | 0.003 | 0.266* | (0.160, 0.372) |  |  | 0.169* | (0.059, 0.278) |  |
| Strengthening Activity (hours/week) | 0.001 | 0.272* | (0.075, 0.469) |  |  | 0.096 | -0.104, 0.295) |  |

*The R^2^ for Block 1 was 0.027. This increased significantly when adding Block 2 (p<0.001).*

**p<0.05*

b) Never smoked

|  | **Crude Associations** | | | **Adjusted Associations** | | |
| --- | --- | --- | --- | --- | --- | --- |
| **Variable** | **R^2^** | **ẞ** | **CI** | **R^2^** | **ẞ** | **CI** |
| Sitting Time (hours/week) | 0.001 | -0.087* | (-0.154, -0.021) | 0.027 | -0.106* | (-0.173, -0.038) |
| Walking (hours/week) | 0.001 | 0.124* | (0.027, 0.221) |  | 0.075 | (-0.023, 0.172) |
| Light Intensity PA (hours/week) | 0.000 | 0.050 | (-0.141, 0.240) |  | -0.047 | (-0.236, 0.142) |
| Moderate Intensity PA (hours/week) | 0.000 | 0.103 | (-0.097, 0.304) |  | 0.019 | (-0.181, 0.218) |
| Strenuous PA (hours/week) | 0.002 | 0.213* | (0.073, 0.352) |  | 0.194* | (0.050, 0.339) |
| Strengthening Activity (hours/week) | 0.000 | 0.160 | (-0.114, 0.435) |  | -0.005 | (-0.284, 0.273) |

*The R^2^ for Block 1 was 0.022. This increased significantly when adding Block 2 (p=0.002).*

**p<0.05*

c) Less than 10 pack years

|  | **Crude Associations** | | | **Adjusted Associations** | | |
| --- | --- | --- | --- | --- | --- | --- |
| **Variable** | **R^2^** | **ẞ** | **CI** | **R^2^** | **ẞ** | **CI** |
| Sitting Time (hours/week) | 0.000 | 0.038 | (-0.053, 0.130) | 0.030 | 0.068 | (-0.024, 0.161) |
| Walking (hours/week) | 0.006 | 0.248* | (0.123, 0.373) |  | 0.176* | (0.049, 0.303) |
| Light Intensity PA (hours/week) | 0.005 | 0.456* | (0.191, 0.720) |  | 0.370* | (0.106, 0.634) |
| Moderate Intensity PA (hours/week) | 0.000 | 0.143 | (-0.119, 0.404) |  | 0.060 | (-0.199, 0.320) |
| Strenuous PA (hours/week) | 0.001 | 0.174 | (-0.007, 0.356) |  | 0.059 | (-0.128, 0.246) |
| Strengthening Activity (hours/week) | 0.003 | 0.434* | (0.103, 0.766) |  | 0.303 | (-0.032, 0.638) |

*The R^2^ for Block 1 was 0.020. This increased significantly when adding Block 2 (p=0.001).*

**p<0.05*

d) More than 10 pack years

|  | **Crude Associations** | | | **Adjusted Associations** | | |
| --- | --- | --- | --- | --- | --- | --- |
| **Variable** | **R^2^** | **ẞ** | **CI** | **R^2^** | **ẞ** | **CI** |
| Sitting Time (hours/week) | 0.000 | -0.012 | (-0.133, 0.110) | 0.008 | 0.013 | (-0.112, 0.138) |
| Walking (hours/week) | 0.000 | 0.066 | (-0.099, 0.230) |  | 0.017 | (-0.152, 0.186) |
| Light Intensity PA (hours/week) | 0.000 | 0.055 | (-0.238, 0.349) |  | 0.035 | (-0.263, 0.333) |
| Moderate Intensity PA (hours/week) | 0.000 | 0.093 | (-0.230, 0.415) |  | 0.047 | (-0.282, 0.376) |
| Strenuous PA (hours/week) | 0.002 | 0.285 | (-0.035, 0.605) |  | 0.237 | (-0.098, 0.572) |
| Strengthening Activity (hours/week) | 0.000 | 0.077 | (-0.415, 0.570) |  | -0.090 | (-0.600, 0.420) |

*The R^2^ for Block 1 was 0.007. This did not increase significantly when adding Block 2 (p=0.895).*

**p<0.05*
