# Supplementary material for: Movement behaviours are associated with lung function in middle-aged and older adults: a cross-sectional analysis of the Canadian longitudinal study on aging
Source: BMC Public Health. 2018 Jul 3;18:818. doi: 10.1186/s12889-018-5739-4 (PMC6029121; doi:10.1186/s12889-018-5739-4)
Supplement: Supplementary file 3 — Table S3. Association between movement behaviours and %predicted FVC by smoking history among males. (DOCX 22 kb) [file 12889_2018_5739_MOESM3_ESM.docx]

Additional file

Table S3: Association between movement behaviours and %predicted FVC by smoking history among males

a) All Smoker Types

|  | **Crude Associations** | | | | **Adjusted Associations** | | | |
| --- | --- | --- | --- | --- | --- | --- | --- | --- |
| **Variable** | **R^2^** | **ẞ** | **CI** | **R^2^** | | **ẞ** | **CI** |  |
| Sitting Time (hours/week) | 0.009 | -0.198* | (-0.240, -0.155) | 0.120 | | -0.059* | (-0.101, -0.018) |  |
| Walking (hours/week) | 0.003 | 0.153* | (0.096, 0.209) |  |  | 0.099* | (0.045, 0.153) |  |
| Light Intensity PA (hours/week) | 0.000 | 0.084 | (-0.014, 0.181) |  |  | 0.102* | (0.010, 0.195) |  |
| Moderate Intensity PA (hours/week) | 0.001 | 0.118* | (0.031, 0.205) |  |  | 0.081 | (-0.001, 0.164) |  |
| Strenuous PA (hours/week) | 0.017 | 0.458* | (0.384, 0.531) |  |  | 0.231* | (0.159, 0.303) |  |
| Strengthening Activity (hours/week) | 0.005 | 0.491* | (0.346, 0.637) |  |  | 0.247* | (0.107, 0.387) |  |

*The R^2^ for Block 1 was 0.109. This increased significantly when adding Block 2 (p<0.001).*

**p<0.05*

b) Never smoked

|  | **Crude Associations** | | | **Adjusted Associations** | | |
| --- | --- | --- | --- | --- | --- | --- |
| **Variable** | **R^2^** | **ẞ** | **CI** | **R^2^** | **ẞ** | **CI** |
| Sitting Time (hours/week) | 0.009 | -0.183* | (-0.241, -0.126) | 0.106 | -0.064* | (-0.120, -0.008) |
| Walking (hours/week) | 0.001 | 0.099* | (0.018, 0.180) |  | 0.058 | (-0.019, 0.136) |
| Light Intensity PA (hours/week) | 0.000 | 0.035 | (-0.106, 0.176) |  | 0.067 | (-0.068, 0.202) |
| Moderate Intensity PA (hours/week) | 0.000 | 0.069 | (-0.060, 0.198) |  | 0.076 | (-0.048, 0.199) |
| Strenuous PA (hours/week) | 0.024 | 0.544* | (0.442, 0.647) |  | 0.353* | (0.252, 0.454) |
| Strengthening Activity (hours/week) | 0.003 | 0.369* | (0.159, 0.578) |  | 0.119 | (-0.082, 0.321) |

*The R^2^ for Block 1 was 0.092. This increased significantly when adding Block 2 (p<0.001).*

**p<0.05*

c) Less than 10 pack years

|  | **Crude Associations** | | | **Adjusted Associations** | | |
| --- | --- | --- | --- | --- | --- | --- |
| **Variable** | **R^2^** | **ẞ** | **CI** | **R^2^** | **ẞ** | **CI** |
| Sitting Time (hours/week) | 0.006 | -0.150* | (-0.233, -0.067) | 0.088 | -0.047 | (-0.128, 0.034) |
| Walking (hours/week) | 0.000 | 0.011 | (-0.098, 0.119) |  | -0.029 | (-0.135, 0.077) |
| Light Intensity PA (hours/week) | 0.001 | 0.114 | (-0.061, 0.289) |  | 0.128 | (-0.043, 0.298) |
| Moderate Intensity PA (hours/week) | 0.000 | -0.034 | (-0.185, 0.118) |  | -0.112 | (-0.260, 0.037) |
| Strenuous PA (hours/week) | 0.015 | 0.397 | (0.260, 0.535) |  | 0.229* | (0.089, 0.368) |
| Strengthening Activity (hours/week) | 0.005 | 0.411 | (0.160, 0.661) |  | 0.271* | (0.016, 0.526) |

*The R^2^ for Block 1 was 0.078. This increased significantly when adding Block 2 (p<0.001).*

**p<0.05*

d) More than 10 pack years

|  | **Crude Associations** | | | **Adjusted Associations** | | |
| --- | --- | --- | --- | --- | --- | --- |
| **Variable** | **R^2^** | **ẞ** | **CI** | **R^2^** | **ẞ** | **CI** |
| Sitting Time (hours/week) | 0.006 | -0.170* | (-0.266, -0.073) | 0.145 | -0.033 | (-0.125, 0.059) |
| Walking (hours/week) | 0.021 | 0.379* | (0.268, 0.489) |  | 0.269* | (0.163, 0.375) |
| Light Intensity PA (hours/week) | 0.001 | 0.146 | (-0.053, 0.345) |  | 0.182 | (-0.004, 0.369) |
| Moderate Intensity PA (hours/week) | 0.008 | 0.358* | (0.184, 0.532) |  | 0.294* | (0.130, 0.457) |
| Strenuous PA (hours/week) | 0.003 | 0.190* | (0.034, 0.345) |  | -0.035 | (-0.184, 0.114) |
| Strengthening Activity (hours/week) | 0.008 | 0.650* | (0.336, 0.965) |  | 0.473* | (0.172, 0.774) |

*The R^2^ for Block 1 was 0.120. This increased significantly when adding Block 2 (p<0.001).*

**p<0.05*
