# Supplementary material for: Movement behaviours are associated with lung function in middle-aged and older adults: a cross-sectional analysis of the Canadian longitudinal study on aging
Source: BMC Public Health. 2018 Jul 3;18:818. doi: 10.1186/s12889-018-5739-4 (PMC6029121; doi:10.1186/s12889-018-5739-4)
Supplement: Supplementary file 4 — Table S4. Association between movement behaviours and %predicted FVC by smoking history among females. (DOCX 22 kb) [file 12889_2018_5739_MOESM4_ESM.docx]

Additional file

Table S4: Association between movement behaviours and %predicted FVC by smoking history among females

a) All Smoker Types

|  | **Crude Associations** | | | | **Adjusted Associations** | | | |
| --- | --- | --- | --- | --- | --- | --- | --- | --- |
| **Variable** | **R^2^** | **ẞ** | **CI** | **R^2^** | | **ẞ** | **CI** |  |
| Sitting Time (hours/week) | 0.006 | -0.162* | (-0.206, -0.119) | 0.068 | | -0.051* | (-0.094, -0.007) |  |
| Walking (hours/week) | 0.004 | 0.193* | (0.131, 0.255) |  |  | 0.093* | (0.031, 0.154) |  |
| Light Intensity PA (hours/week) | 0.000 | 0.108 | (-0.014, 0.229) |  |  | 0.049 | (-0.069, 0.168) |  |
| Moderate Intensity PA (hours/week) | 0.001 | 0.197* | (0.070, 0.324) |  |  | 0.087 | (-0.037, 0.211) |  |
| Strenuous PA (hours/week) | 0.008 | 0.389* | (0.296, 0.482) |  |  | 0.145* | (0.050, 0.239) |  |
| Strengthening Activity (hours/week) | 0.002 | 0.364* | (0.190, 0.537) |  |  | 0.084 | (-0.088, 0.257) |  |

*The R^2^ for Block 1 was 0.064. This increased significantly when adding Block 2 (p<0.001).*

**p<0.05*

b) Never smoked

|  | **Crude Associations** | | | **Adjusted Associations** | | |
| --- | --- | --- | --- | --- | --- | --- |
| **Variable** | **R^2^** | **ẞ** | **CI** | **R^2^** | **ẞ** | **CI** |
| Sitting Time (hours/week) | 0.009 | -0.194* | (-0.254, -0.134) | 0.056 | -0.103* | (-0.163, -0.043) |
| Walking (hours/week) | 0.003 | 0.174* | (0.086, 0.261) |  | 0.075 | (-0.012, 0.162) |
| Light Intensity PA (hours/week) | 0.000 | 0.062 | (-0.110, 0.234) |  | -0.001 | (-0.169, 0.167) |
| Moderate Intensity PA (hours/week) | 0.001 | 0.217* | (0.037, 0.398) |  | 0.106 | (-0.071, 0.283) |
| Strenuous PA (hours/week) | 0.009 | 0.407* | (0.282, 0.533) |  | 0.195* | (0.066, 0.323) |
| Strengthening Activity (hours/week) | 0.001 | 0.264* | (0.016, 0.511) |  | -0.034 | (-0.281, 0.213) |

*The R^2^ for Block 1 was 0.051. This increased significantly when adding Block 2 (p<0.001).*

**p<0.05*

c) Less than 10 pack years

|  | **Crude Associations** | | | **Adjusted Associations** | | |
| --- | --- | --- | --- | --- | --- | --- |
| **Variable** | **R^2^** | **ẞ** | **CI** | **R^2^** | **ẞ** | **CI** |
| Sitting Time (hours/week) | 0.002 | -0.091* | (-0.171, -0.011) | 0.083 | 0.034 | (-0.045, 0.114) |
| Walking (hours/week) | 0.009 | 0.258* | (0.148, 0.367) |  | 0.172* | (0.063, 0.281) |
| Light Intensity PA (hours/week) | 0.003 | 0.330* | (0.097, 0.563) |  | 0.250* | (0.024, 0.476) |
| Moderate Intensity PA (hours/week) | 0.001 | 0.220 | (-0.010, 0.450) |  | 0.115 | (-0.107, 0.337) |
| Strenuous PA (hours/week) | 0.005 | 0.272* | (0.112, 0.431) |  | 0.031 | (-0.130, 0.191) |
| Strengthening Activity (hours/week) | 0.005 | 0.533* | (0.241, 0.825) |  | 0.291* | (0.004, 0.578) |

*The R^2^ for Block 1 was 0.074. This increased significantly when adding Block 2 (p=0.001).*

**p<0.05*

d) More than 10 pack years

|  | **Crude Associations** | | | **Adjusted Associations** | | |
| --- | --- | --- | --- | --- | --- | --- |
| **Variable** | **R^2^** | **ẞ** | **CI** | **R^2^** | **ẞ** | **CI** |
| Sitting Time (hours/week) | 0.002 | -0.095 | (-0.199, 0.008) | 0.075 | -0.004 | (-0.106, 0.099) |
| Walking (hours/week) | 0.001 | 0.112 | (-0.028, 0.251) |  | 0.001 | (-0.138, 0.139) |
| Light Intensity PA (hours/week) | 0.000 | -0.008 | (-0.257, 0.241) |  | -0.020 | (-0.264, 0.224) |
| Moderate Intensity PA (hours/week) | 0.000 | 0.116 | (-0.158, 0.389) |  | 0.064 | (-0.205, 0.334) |
| Strenuous PA (hours/week) | 0.003 | 0.296* | (0.025, 0.568) |  | 0.127 | (-0.147, 0.402) |
| Strengthening Activity (hours/week) | 0.000 | 0.172 | (-0.246, 0.589) |  | -0.014 | (-0.431, 0.404) |

*The R^2^ for Block 1 was 0.075. This did not increase significantly when adding Block 2 (p=0.980).*

**p<0.05*
